# Supplementary figures and images for: Spt5 C-terminal repeat domain phosphorylation and length negatively regulate heterochromatin through distinct mechanisms
Source: PLoS Genet. 2023 Nov 8;19(11):e1010492. doi: 10.1371/journal.pgen.1010492 (PMC10659198; doi:10.1371/journal.pgen.1010492)

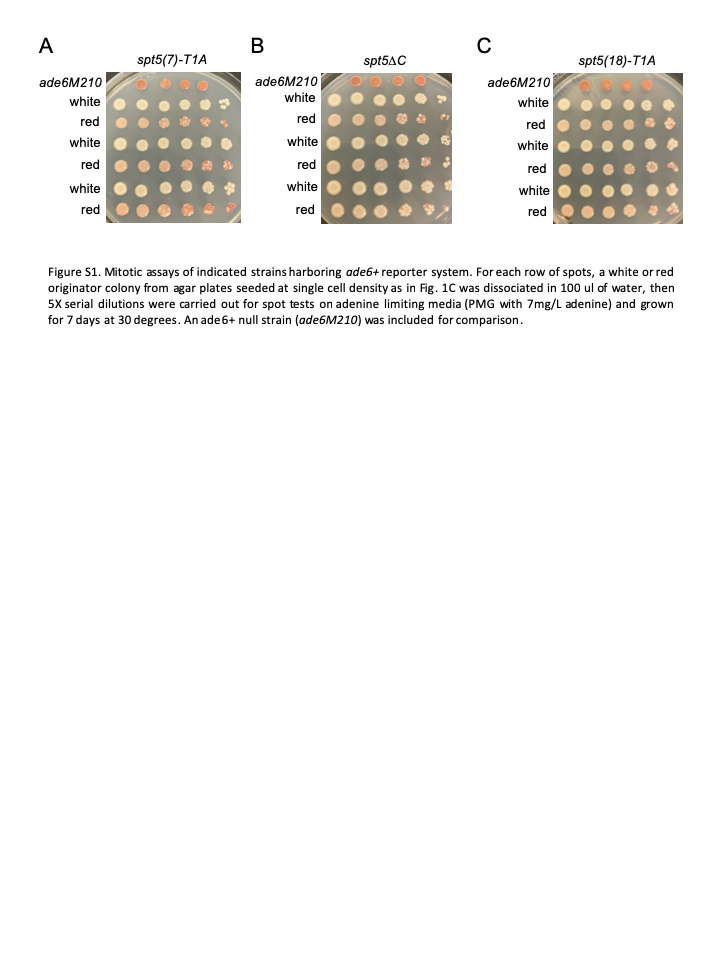

Supplement: S1 Fig — For each row of spots, a white or red originator colony from agar plates seeded at single cell density as in Fig 1C was dissociated in 100 ul of water, then 5X serial dilutions were carried out for spot tests on adenine limiting media (PMG with 7mg/L adenine) and grown for 7 days at 30 degrees. An ade6 mutant (ade6-M210) was included for comparison. (TIFF) [file pgen.1010492.s001.tiff]

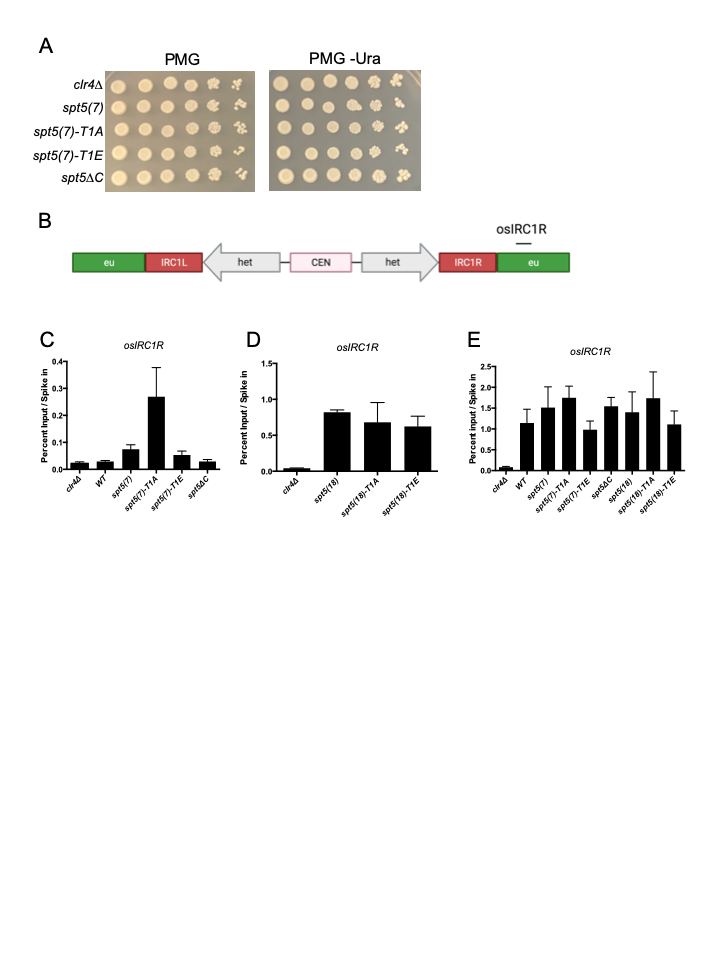

Supplement: S2 Fig — (A) Spot tests of indicated spt5 mutants on control PMG media and PMG lacking uracil. Plates were incubated at 30 degrees for 3 days before imaging. (B) Anti-H3K9me2 ChIP-qPCR in indicated spt5 mutants and controls. Primers amplify an intergenic region <1 kb outside of the irc1R boundary (osIRC1R). Percent input was normalized to mouse spike-in. clr4Δ was included as a negative control. Error bars indicate SEM (n = 3). (C and D) As in (B) for anti-H3K9me3 ChIP-qPCR. Percent input was normalized to S. cerevisiae spike-in. (TIFF) [file pgen.1010492.s002.tiff]

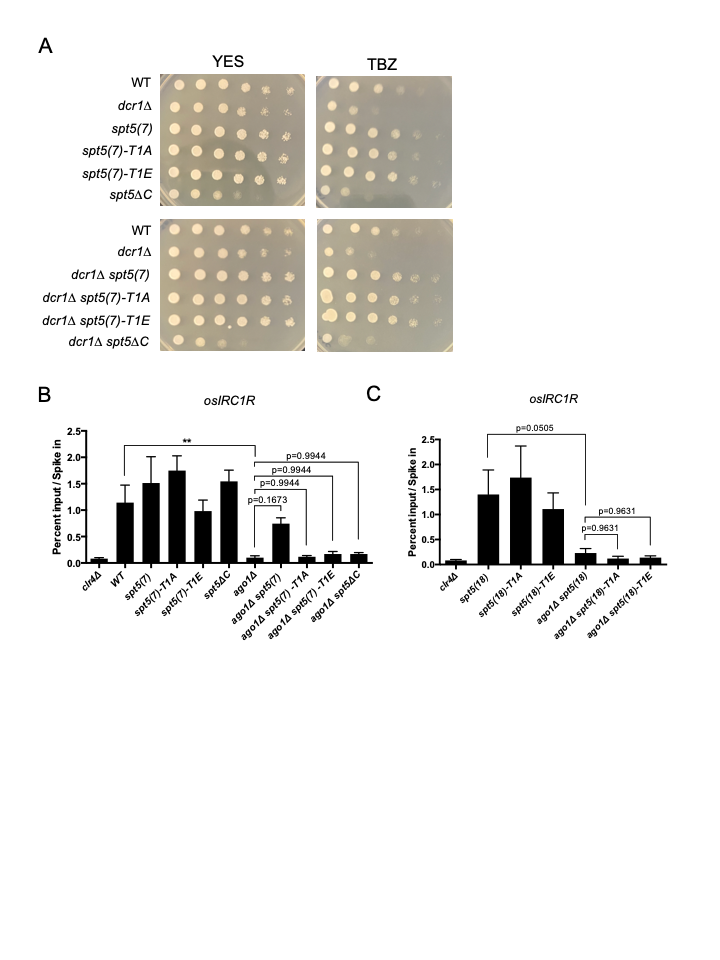

Supplement: S3 Fig — (A) Spot tests of the indicated strains on control media (YES) or YES containing thiabendazole (TBZ). Plates were incubated for 3 days at 30 degrees before imaging. (B and C) Anti-H3K9me3 ChIP-qPCR quantified with osIRC1R primers in the indicated strains. Percent input was normalized to S. cerevisiae spike-in. Error bars indicate SEM. Asterisks indicate significant differences between indicated comparisons [p<0.05 for 1 star, p<0.01 for 2 stars, p<0.001 for 3 stars, one-way ANOVA followed by Holm-Sidak’s multiple comparison test (unpaired) with a single pooled variance; n = 3). (TIFF) [file pgen.1010492.s003.tiff]

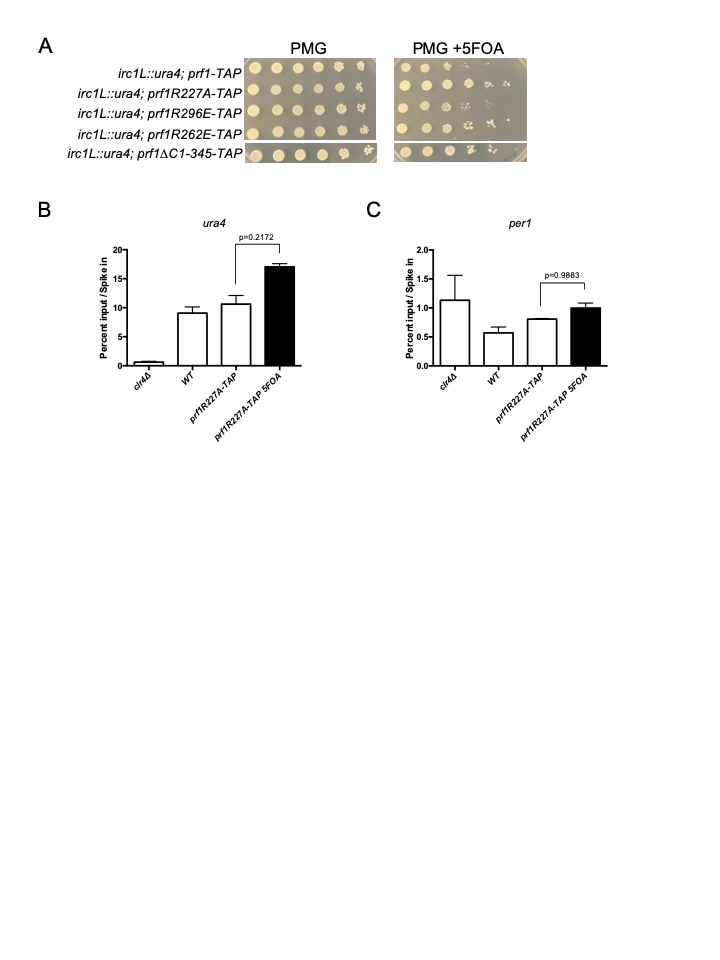

Supplement: S4 Fig — (A) Spot tests of indicated prf1 mutants on control PMG media and PMG with 5’FOA. Plates were incubated at 30 degrees for 7 days before imaging. (B and C) Anti-H3K9me3 ChIP-qPCR carried out as in Fig 2 using cells of the indicated genotypes grown in non-selective media (white bars) or 5’FOA-containing media (black bars); clr4Δ was included as a negative control. Percent input was normalized to S. cerevisiae spike-in. Error bars indicate SEM. Asterisks indicate significant differences for the indicated comparisons [p<0.05, one-way ANOVA followed by Holm-Sidak’s multiple comparison test (unpaired) with a single pooled variance; n = 3]. (TIFF) [file pgen.1010492.s004.tiff]
