# Supplementary material for: Spt5 C-terminal repeat domain phosphorylation and length negatively regulate heterochromatin through distinct mechanisms
Source: PLoS Genet. 2023 Nov 8;19(11):e1010492. doi: 10.1371/journal.pgen.1010492 (PMC10659198; doi:10.1371/journal.pgen.1010492)
Supplement: S1 Table — (DOCX) [file pgen.1010492.s005.docx]

**S1_Table**. Summary of *spt5^+^* alleles used in this study.

| **Notation** | **Description of Spt5 CTD** | **Additional marker at *spt5* locus** |
| --- | --- | --- |
| WT | 18 repeats of motif [T_1_PAWNSGSK] | N/A |
| *spt5(18)* | 18 repeats of motif [T_1_PAWNSGSK] | *ura4^+^* or *natMX6^+^* |
| *spt5(18)-T1A* | 18 repeats of motif with threonine 1 mutated to alanine [A_1_PAWNSGSK] | *ura4^+^* or *natMX6^+^* |
| *spt5(18)-T1E* | 18 repeats of motif with threonine 1 mutated to glutamate [E_1_PAWNSGSK] | *ura4^+^* or *natMX6^+^* |
| *spt5(7)* | 7 repeats of motif [T_1_PAWNSGSK] | *ura4^+^* or *natMX6^+^* |
| *spt5(7)-T1A* | 7 repeats of motif with threonine 1 mutated to alanine [A_1_PAWNSGSK] | *ura4^+^* or *natMX6^+^* |
| *spt5(7)-T1E* | 7 repeats of motif with threonine 1 mutated to glutamate [E_1_PAWNSGSK] | *ura4^+^* or *natMX6^+^* |
| *spt5ΔC* | C-terminal deletion to residue 880 | *ura4^+^* or *kanMX6^+^* |
