# Supplementary material for: Spt5 C-terminal repeat domain phosphorylation and length negatively regulate heterochromatin through distinct mechanisms
Source: PLoS Genet. 2023 Nov 8;19(11):e1010492. doi: 10.1371/journal.pgen.1010492 (PMC10659198; doi:10.1371/journal.pgen.1010492)
Supplement: S2 Table — (DOCX) [file pgen.1010492.s006.docx]

**S2_Table.** *S. pombe* strains used in this study.

| Strain | Genotype | Source |
| --- | --- | --- |
| JT362 | *h+ leu1-32 ura4-D18 his3-D1 ade6-M210* | (56) |
| JT249 | *h+ leu1-32 ade6-M210 ura4DS/E otr1R(SphI)::ura4 oriA clr4Δ::kanmx6* | D. Moazed |
| JT340 | *h- spt5(7)::ura4+ leu1-32 ura4-D18 his3-D1 ade6-M210* | B. Schwer |
| JT341 | *h- spt5(7)-T1A::ura4+ leu1-32 ura4-D18 his3-D1 ade6+* | B. Schwer |
| JT342 | *h- spt5(7)-T1E::ura4+ leu1-32 ura4-D18 his3-D1 ade6+* | B. Schwer |
| JT343 | *h- spt5ΔC::ura4+ leu1-32 ura4-D18 his3-D1 ade6+* | B. Schwer |
| JT866 | *h? irc1L::ura4+ spt5ΔC::kanmx6 leu? ura4D-18 ade6?* | This study |
| JT871 | *h? spt5ΔC::kanMx6 leu1+ ura4+ ade6?* | (56) |
| JT921 | *h- prf1ΔC(1-345)-TAP::kanmx6 ade6-M216* | (68) |
| JT924 | *﻿h- prf1-R227A-TAP::kanMX6 ade6-M216* | (68) |
| JT926 | *﻿h- prf1-R262E-TAP::kanMX6 ade6-M216* | (68) |
| JT202 | *﻿h- prf1-TAP::kanMX6* | (56) |
| JT793 | *h- IRC1L (Xho1):ura4+ per1::NatR cycR? ade6-210 leu1-32 ura4-D18* | (66) |
| JT943 | *h- ago1Δ::hph leu1-32 ura4-UTRΔ::kanmx6 ade6-M210* | D. Moazed |
| JT944 | *h+ otr1R(SphI)::ade6+ leu1-32 ura4-D18* | D. Moazed |
| JT972 | *h? otr1R(SphI)::ade6+ spt5(7)::ura4+ leu? ura4D18* | This study |
| JT973 | *h? otr1R(SphI)::ade6+ spt5(7)-T1A::ura4+ leu? ura4D18* | This study |
| JT974 | *h? otr1R(SphI)::ade6+ spt5(7)-T1E::ura4+ leu? ura4D18* | This study |
| JT975 | *h? otr1R(SphI)::ade6+ spt5ΔC::ura4+ leu? ura4D18* | This study |
| JT991 | *h? ago1delta::hphMX6 spt5(7)::ura4+* | This study |
| JT992 | *h? ago1delta::hph spt5(7)-T1A::ura4+* | This study |
| JT993 | *h? ago1delta::hph spt5(7)-T1E::ura4+* | This study |
| JT994 | *h? ago1delta::hph spt5ΔC::ura4+* | This study |
| JT1030 | *h- irc1L(Xho1):ura4+ per1:hph* | This study |
| JT1034 | *h? irc1L(Xho1):ura4+ per1:hph spt5(7)::natmx6* | This study |
| JT1035 | *h? irc1L(Xho1):ura4+ per1:hph spt5(7)-T1A::natmx6* | This study |
| JT1036 | *h? irc1L(Xho1):ura4+ per1:hph spt5(7)-T1E::natmx6* | This study |
| JT1047 | *h- spt5(18)::ura4+ leu1-32 ura4-D18 his3-D1 ade6-M210* | B. Schwer |
| JT1048 | *h- spt5(18)-T1A::ura4+ leu1-32 ura4-D18 his3-D1 ade6-M210* | B. Schwer |
| JT1049 | *h- spt5(18)-T1E::ura4+ leu1-32 ura4-D18 his3-D1 ade6-M210* | B. Schwer |
| JT1071 | *h? spt5(7)::natmx6 leu1-32 ura4-D18 his3-D1 ade6-M210* | This study |
| JT1072 | *h? spt5(7)-T1A::natmx6 leu1-32 ura4-D18 his3-D1 ade6-M210* | This study |
| JT1073 | *h? spt5(7)-T1E::natmx6 leu1-32 ura4-D18 his3-D1 ade6-M210* | This study |
| JT1075 | *h? otr1R(SphI)::ade6+ prf1-TAP::kanmx6* | This study |
| JT1076 | *h? otr1R(SphI)::ade6+ prf1R227A-TAP::kanmx6* | This study |
| JT1077 | *h? irc1L(Xho1)::ura4+ per1::hphMX6 prf1-TAP::kanMX6 ura4-D18* | This study |
| JT1104 | *h? irc1L(Xho1):ura4+ per1::hphMX6 prf1R227A-TAP::kanMX6 ura4-D18* | This study |
| JT1105 | *h? irc1L(Xho1):ura4+ per1::hphMX6 prf1ΔC(1-345)-TAP::kanMX6 ura4-D18* | This study |
| JT1131 | *h? otr1R(SphI)::ade6+ spt5(18)::ura4+* | This study |
| JT1132 | *h? otr1R(SphI)::ade6+ spt5(18)-T1A::ura4+* | This study |
| JT1133 | *h? otr1R(SphI)::ade6+ spt5(18)-T1E::ura4+* | This study |
| JT1141 | *h? otr1R(SphI)::ade6+ prf1R296E::kanmx6* | This study |
| JT1142 | *h? otr1R(SphI)::ade6+ prf1R262E::kanmx6* | This study |
| JT1171 | *h? ago1Δ::hph spt5(18)::ura4+* | This study |
| JT1172 | *h? ago1Δ::hph spt5(18)-T1A::ura4+* | This study |
| JT1192 | *h+ spt5(18)::natMX6 leu1-32 ura4-D18 his3-D1 ade6-M210* | This study |
| JT1193 | *h+ spt5(18)-T1A::natMX6 leu1-32 ura4-D18 his3-D1 ade6-M210* | This study |
| JT1194 | *h+ spt5(18)-T1E::natMX6 leu1-32 ura4-D18 his3-D1 ade6-M210* | This study |
| JT1247 | *h? ago1Δ::hphMX6 spt5(18)-T1E::ura4+* | This study |
| JT1174 | *h? dcr1Δ::hphMX6 spt5(7)::ura4+* | This study |
| JT1178 | *h? dcr1Δ::hphMX6 spt5(7)-T1A::ura4+* | This study |
| JT1179 | *h? dcr1Δ::hphMX6 spt5(7)-T1E::ura4+* | This study |
| JT1180 | *h? dcr1Δ::hphMX6 spt5ΔC::ura4+* | This study |
| JT1248 | *h? irc1L(Xho1):ura4+ per1::hphMX6 prf1R296E-TAP::kanMX6 ura4-D18* | This study |
| JT1245 | *h? irc1L(Xho1):ura4+ per1::hphMX6 prf1R262E-TAP::kanMX6 ura4-D18* | This study |
| JT1308 | *h? ago1Δ::hphMX6 prf1R262E-TAP* | This study |
| JT1309 | *h? ago1Δ::hphMX6 prf1ΔC(1-345)-TAP::kanMX6* | This study |
| JT1310 | *h? ago1Δ::hphMX6 prf1-TAP::kanmx6* | This study |
| JT1315 | *h? ago1Δ::hphMX6 prf1R227A-TAP::kanmx6* | This study |
| JT1200 | *h- irc1L(Xho1):ura4+ per1::hphMX6 spt5(18)::ura4+* | This study |
| JT1201 | *h- irc1L(Xho1):ura4+ per1::hphMX6 spt5(18)-T1A::ura4+* | This study |
| JT1386 | h? *otr1R(SphI)::ade6+* *spt5(7)-T1A::ura4+* *prf1-TAP::kanMX6* | This study |
| JT1387 | h? *otr1R(SphI)::ade6+* *spt5(7)-T1A::ura4+ prf1R227A-TAP::kanMX6* | This study |
| JT1390 | h? *otr1R(SphI)::ade6+* *spt5(18)-T1A::ura4+ prf1-TAP::kanMX6* | This study |
| JT1391 | h? *otr1R(SphI)::ade6+* *spt5(18)-T1A::ura4+ prf1R227A-TAP::kanMX6* | This study |
| JT997 | ﻿*h? prf1-R227A-TAP::kanMX6 spt5(7)::ura4 ade6-M216 ura4-D18 leu1-32? his3-D1?* | (68) |
| JT998 | ﻿*h? prf1-R227A-TAP::kanMX6 spt5-T1A(7)::ura4 ade6-M216 ura4-D18 leu1-32? his3-D1?* | (68) |
| JT1107 | *h? spt5(18)::ura4+ prf1R227A-TAP::kanMX6 ura4-D18* | This study |
| JT1108 | *h? spt5(18)::ura4+ prf1R227A-TAP::kanMX6 ura4-D18* | This study |
