# Supplementary material for: Spt5 C-terminal repeat domain phosphorylation and length negatively regulate heterochromatin through distinct mechanisms
Source: PLoS Genet. 2023 Nov 8;19(11):e1010492. doi: 10.1371/journal.pgen.1010492 (PMC10659198; doi:10.1371/journal.pgen.1010492)
Supplement: S3 Table — (DOCX) [file pgen.1010492.s007.docx]

**S3_Table**. Oligonucleotide primers used in this study.

| Primer | Sequence |
| --- | --- |
| Vtc4’Fw | AGCCAAACATAATGCGGTCC |
| Vtc4’Rv | AACATTGGCGCTGATTGCAG |
| Dh’Fw | ACAATTGTGACTTTGTTGGT |
| Dh’Rv | TGTCCATACCCATGCTGTGTC |
| OsIRC1R’Fw | GAGAGGTTTCTTATCGTGCTATC |
| OsIRC1R’Rv | GAGTGTAAACTTAGTGTGAGCG |
| Pma1’Fw | ACCCCAGCTAGTTAAAGAAAATCA |
| Pma1’Rv | CGTCATCGTCAGAAGATTCAGATG |
| Pou5F’Fw | GTGGGTAAGCAAGAACTGAGGA |
| Pou5F’Rv | TGGAGAGCCTAAAACATCCATT |
| Ade6’Fw | GCAGGCCAAGAGTTTGGTTAT |
| Ade6’Rv | CCAAGTGCTTTGATGGCAGTA |
| Act1’Fw | CCACTATGTATCCCGGTATTGC |
| Act1’Rv | CAATCTTGACCTTCATGGAGCT |
| Dg’Fw | ACAATTAGGGCATGTGGTGT |
| Markerswitch’Fw | CGGATCCCCGGGTTAATTAAGGCG |
| Markerswitch’Rv | GAATTCGAGCTCATTTAAACACTGGATG |
| Spt5Ura4’Fw | GGCTGGGACAGCAATATCGT |
| Spt5Ura4’Rv | GCTCCATAGACTCCACGACC |
| Per1’Fw | CCTCGTGCTACCAAACAGGT |
| Per1’Rv | ACCATCGCTGCTATTACCCA |
