# Supplementary material for: Spt5 C-terminal repeat domain phosphorylation and length negatively regulate heterochromatin through distinct mechanisms
Source: PLoS Genet. 2023 Nov 8;19(11):e1010492. doi: 10.1371/journal.pgen.1010492 (PMC10659198; doi:10.1371/journal.pgen.1010492)
Supplement: Attachment — Submitted filename: Response to Reviewer Comments_Aug2023.pdf [file pgen.1010492.s008.pdf]

Response to Reviewer Comments

We thank the reviewers for their comments and suggestions. In response to their feedback, we clarified the heterochromatin spreading phenotype in *spt5-T1A* and *prf1* mutant strains using H3K9me3 ChIP analyses (updated Figure 2 and Figure S4). We also clarified rescue of *ago1Δ* by *prf1* mutants with H3K9me3 ChIP and RT-qPCR (see updated Figure 5). Finally, we assessed aberrant heterochromatin formation in *spt5 prf1* double mutants; these results revealed a synthetic effect modulated by Spt5 CTD repeat number (Figure 6). Overall, the new data strengthen our conclusion that the length of the Spt5 CTD array and its phosphorylation regulate heterochromatin through different mechanisms. Please find our point-by-point responses to the reviewer comments below (our responses in red).

Reviewer 1:

The observation of the separation of function (prevention of RNAi-dependent and independent heterochromatin) of Spt5-CTD is interesting as the authors suggested. However, it is easy to expect that Spt5-CTD prevents the aberrant targeting of RNAi machinery to an ectopic site, because the previous results showed loss Spt5-CTD interacting factor Rtf1 caused the aberrant targeting.

Our results extend the previously published work on Prf1/Rtf1 by providing a detailed analysis of the effects of the Spt5 CTD on heterochromatin. We demonstrate that phosphorylation of the repeats and the size of the repeat array influence heterochromatin differently, likely through differential effects on RNAi-dependent and RNAi-independent pathways. We also illuminate the interplay between the Spt5 CTD and Prf1 in mediating these effects. We think these findings represent a significant advance over previous work.

Moreover, authors only compared the phenotypes of the mutants and made a lot of discussion about the possible mechanisms of Spt5-mediated suppression of heterochromatin formation without concrete results. I think several possibilities raised in the discussion, like involvement of processing of nascent transcripts or transcription elongation speed of RNA polymerase II, should be examined.

Investigation of the mechanisms underlying these phenotypes is a priority for us going forward. We are in the process of testing some of the key hypotheses, but sorting out the specific factors mediating the RNAi-dependent and RNAi-independent effects we observe will take time. We think this work is beyond the scope of what we can include in the present study. Moreover, we think that our comprehensive description of the effects of Spt5 and Prf1 mutants on heterochromatin is of sufficient interest on its own.

1) Page 4, line 1. I think the following papers that describes the involvement of RNA polymerase II in RNAi-dependent heterochromatin formation should be referred.
Kato, Hiroaki, et al. "RNA polymerase II is required for RNAi-dependent heterochromatin assembly." Science 309.5733 (2005): 467-469.

Djupedal, Ingela, et al. "RNA Pol II subunit Rpb7 promotes centromeric transcription and RNAi-directed chromatin silencing." Genes & development 19.19 (2005): 2301-2306.

We agree with the reviewer and have added these references (see last line of page 3).

2) Page 5, line 7. "Prf1/Rtf1" suddenly appeared in the text without explanation what it is. Brief explanation will be helpful for the readers.

We now introduce Prf1/Rtf1 and PAF immediately after the Spt5 CTD at the bottom of page 6.

3) Figure 1. To discuss the targeting of RNAi, it is important to show the mutations do not affect the amounts of siRNAs produced from ade6 gene inserted at centromeric heterochromatin. Without this information, it is hard to discuss the targeting efficiency.

Yu et al, 2018 (ref 33) demonstrated, using the same dual ade6+ reporter we use here, that the inappropriate action of the RNAi pathway at euchromatic locations was not associated with increased siRNA production within heterochromatin. Moreover, Kowalik et al (2015; ref 30) found (using a related reporter system in which the siRNA source was also euchromatic) that aberrant "trans" silencing in enabling mutants was not associated with increased siRNA production from the siRNA source. Earlier work (Iida et al, 2008; ref 31) also showed that abundance of siRNAs produced in trans was not a primary factor in their ability to silence a euchromatic target gene. Thus, while we cannot rule out this possibility, we thought it unlikely that the Spt5 CTD mutants were acting through this mechanism.

4) Page 10, lines 3-6. Authors performed H3K9me-ChIP without selection and did not observe increase of H3K9me. To claim that heterochromatin is really expanded and caused the observed FOA resustant phenotype, it is essential to show increase of H3K9me. Why authors did not perform ChIP assay using FOA selection.

We have now included ChIP-qPCR for H3K9me3 performed on cells subjected to 5'FOA selection, shown in Figures 2D, 2E, and S4. The results show that increased silencing of the *irc1L::ura4+* reporter correlates with increased H3K9me3 at the *ura4+* reporter in the *spt5(7)-T1A* strain, but not in the *spt5(18)-T1A*, *spt5ΔC*, or *prf1R227A-TAP* strain. These results suggest that spreading of authentic heterochromatin beyond the *irc1L* boundary requires both truncation and phospho-ablation of the Spt5 CTD. This may be due to the fact that both RNAi-dependent and RNAi-independent pathways are misregulated in the truncated Spt5 CTD mutants. We speculate that the increased growth on 5'FOA in the other mutants analyzed may result from partially active silencing complexes (perhaps formed by and RNAi-dependent pathway) that can overcome the boundary element without forming bona fide heterochromatin.

5) Fig 2SA-C. Please show the position of primers used in H3K9me-ChIP assay in Fig. 2A.

We describe the genomic location of the primer pairs in the legends to Figure 2 and Figure S2. The sequences are provided in Table 2.

6) Fig. 5C. TBZ sensitivity is an indirect assay to examine the heterochromatin. The defects of the components of kinetochore also caused TBZ sensitivity. Thus, it is essential to examine the H3K9me level to discuss heterochromatin formation.

We agree that the TBZ sensitivity assay is indirect and showed subtle effects that we had difficulty reproducing. We have now replaced this data with ChIP-qPCR for H3K9me3 and RT-qPCR as a more direct assessment of *ago1Δ* suppression by prf1 mutants (see new version of Figure 5). The data show weak *ago1Δ* suppression by mutations in the Plus3 domain, as compared to the strong effects of Spt5 CTD truncation. This strengthens our argument that ago1D suppression is dictated by Spt5 CTD repeat number rather than phosphorylation state.

Reviewer 2:

1. With regards to the role of Spt5 CTD, the authors show that in spt5 CTD-delta ago1-delta double mutant cells the residual H3k9me at the pericentromeric dh repeats is lost (Figure 3B). This suggests that the Spt5 CTD is required for RNAi-independent maintenance of the residual H3K9me. This might be worth mentioning as it points to yet another function of Spt5 in heterochromatin maintenance.

We agree that this is an interesting point. This seems to imply that whereas a truncation of the CTD can enhance RNAi-independent silencing, complete removal of the CTD has the opposite effect. We speculate on the implications of this finding in the revised discussion.

2. Although genome-wide ChIP-seq analysis does not seem essential to support the main conclusions of the paper, it would be interesting to test whether the sub telomeric tlh genes are affected by the various spt5 mutations that the authors have generated.

We agree with the reviewer that this is an interesting question. To look at locus-specific effects on heterochromatin spreading, we would ideally use strains with the *ura4+* reporter inserted beyond the heterochromatin boundary at one of the telomeres. Such strains are not readily available, and we did not think we would have time to construct and characterize them ourselves. We did not detect increased enrichment of subtelomeric regions by qPCR in our 5'FOA selection ChIP experiments (data not shown).

3. In place of (or in addition to) Figure 4C, it might be more helpful to include a cartoon model that summarizes the various roles of Spt5 in transcription

elongation and how these roles relate to different heterochromatin assembly pathways.

We have revised Figure 4C to include a more detailed cartoon model.

Editor's comments:

1. H3K9me levels at the pericentromeric boundaries (Figure 2 and S2B-D) should be re-examined following FOA selection, as the current result is inconclusive. (This seems to be a straight-forward experiment analogous to the red-white selection, as described in Figure 1D).

See our response to reviewer 1, point 4.

2. I further share the concern that TBZ growth assays generate indirect results and should be compared with other heterochromatin features, particularly in Figure 5, where prf1 mutants have not been analyzed by H3K9me levels and changes in pericentromeric transcript abundance.

   See our response to reviewer 1, point 6.

3. H3K9me levels should be examined at other heterochromatin regions, as suggested by Reviewer 2, to assess whether the effects on Spt5-CTD are locus-specific.

   See our response to reviewer 2, point 2.

4. Finally, one of the main conclusions of this study is that Prf1 mediates the effects of the CTD on heterochromatin, suggesting that they act in the same pathway. However, I am missing an epistasis analyzing (for example assessing the percentage of ade6-dependent red colony formation). This is critical, as Prf1 seems to affect heterochromatin also in Spt5-CTD independent manner (at least a pericentromeric heterochromatin).

We thank the editor for raising this important point. To address this, we assessed ectopic silencing of euchromatic *ade6+* in *prf1-R227A spt5-T1A* double mutants (see Figure 6). We tested mutants harboring *spt5-T1A* in the context of a full-length or truncated Spt5 CTD. The mutants expressing *spt5-T1A* with a truncated CTD showed a synthetic effect on *ade6+* silencing when combined with *prf1-R227A*, consistent with our previous results in Chen et al, 2020 (ref 68). Interestingly, this was not the case for *spt5-T1A* in the context of the full-length CTD: here *prf1-R227A* and *spt5-T1A* appeared to be epistatic (Figure 6A). We made similar observations in experiments assessing cell separation defects (as we did previously in Chen et al, 2020) in the same double mutant strains (Figure 6B). This experiment was complicated by the fact that in the 18-repeat

spt5-T1A background, the TAP tag on Prf1 seems to cause a partial loss-of-function. Nonetheless, the data provide evidence that the genetic interaction between *prf1* and *spt5* mutations is influenced by the length of the Spt5 CTD repeat array. We discuss the possible implications of this finding in the revised Discussion.
