# Supplementary material for: Spt5 C-terminal repeat domain phosphorylation and length negatively regulate heterochromatin through distinct mechanisms
Source: PLoS Genet. 2023 Nov 8;19(11):e1010492. doi: 10.1371/journal.pgen.1010492 (PMC10659198; doi:10.1371/journal.pgen.1010492)
Supplement: Attachment — Submitted filename: Response to Reviewer Comments_Oct2023.pdf [file pgen.1010492.s009.pdf]

Response to Reviewer Comments

We thank the reviewers and the editor for their positive comments. As both reviewers 1 and 2 had no further comment and recommended acceptance, we focus on the minor points raised by reviewer 3 and the editor. Please find our point-by-point responses below (our responses in red).

Reviewer 3:

1. The paper makes use of multiple alleles and things get a little confusing at times. The naming scheme is introduced in page 9, and it would be simple enough to introduce each allele individually at that point. In the same vein I found the use of the spt5(18) allele in some figures confusing when used together with WT (Figure 4, S2B). I assume this is the WT allele together with the same marker used to make the other spt5 alleles. Perhaps a table would be useful. Finally, the use of the T1E substitution as a phosphomimetic should be explicitly stated as such; the only explanation of this allele is a later mention of it bearing a constitutive negative charge.

Please see Table 1 in the revised manuscript. We have referred to the T1E allele as a phosphomimetic on page 9 of the revised manuscript.

2. Page 15-16: References to Figure 5D should be 5B and 5E should be 5C.

We have fixed these errors.

3. In figure 4B the authors show the recovery of H3K9me3 over dh in ago1D mutants by spt5(7) and spt5(7)T1E, showing that the ago1D mutation does not significantly decrease H3K9me3 levels in these mutants. Wouldn't the correct comparison be ago1D vs ago1D spt5mutant, rather than spt5mutant vs ago1 spt5mutant?

From the context of the comment we assume the reviewer was referring to Figure 3B. We agree with the reviewer and have altered our statistical analyses accordingly. We have made similar changes to Figures 4B, 5B, and S3B-C. The re-analysis has not changed our interpretation of Figure 3B or 4B. There was a small change to our interpretation of Figure 5B, which now supports weak suppression of *ago1*Δ by the Plus3 domain mutations. We have accommodated this change in our description of Figure 5B and in the Discussion.

4. In Figure 5B the differences in dh H3K9me3 between prf1-TAP and prf1-TAP ago1D are marked as significant, but the text (page 15) explicitly states them as non-significant. Same for prf1-R227A-TAP ago1D. Since the effect being assayed is the rescue of H3K9me3 in ago1D by the prf1 mutations (see my previous point) this renders this experiment extremely confusing. Correct the figure or the interpretation.

This has been corrected.

5. The differential effects of these alleles in the forward and reverse dh transcripts is interesting, as it harkens back to observations as old as the original description of RNAi regulation of heterochromatin in Volpe et al 2001, where they showed by run-on assays using swi6D mutants that the forward strand is transcriptionally silenced in WT but the reverse strand is always expressed. The differential regulation of the forward and reverse promoters remains to be elucidated, but Hiten Madhani showed that it could be related to the mechanisms that control nucleosome occupancy at these promoters (Garcia et al G&D 2010). A brief discussion of the potential interaction of the spt6/rtf1 with these mechanisms would be valuable. Could the authors comment on this?

We have not addressed this point in our revisions given that this was not considered a priority by the editor (see below). There is little data available to inform discussion of what the impact of spt5 or prf1 mutations would be on nucleosome occupancy in promoters.

Editor's comments:

The manuscript was fully evaluated at the editorial level and by independent peer reviewers. In addition to the two previous reviewers, the manuscript was also assessed by a third reviewer. All reviewers appreciated the attention to an important topic and were satisfied with the responses. However, the additional reviewer identified some concerns that we ask you address in a revised manuscript.

In particular, this reviewer raised concerns about the control group(s) for comparing the rescue of the ago1 mutant in the double mutants and noted some discrepancies between statements in the text and figures regarding statistical significance (points 3 and 4). In this regard, I also noted that the use of the comparison group (ago1 or spt5/prf1 single mutant) was not always consistent (e.g., in Figures 3B and 4B, the respective spt5 single mutant was used for stastical comparison; in 3C, the ago1 single mutant was used for the comparison). I agree with the reviewer that the direct comparison between ago1 and the double mutant is more informative than the comparison between the single and double spt5 mutants.

We have addressed these points above.

One the same line, I would also suggest to consider whether statistical comparisons for WT and spt5 mutants (with and without FOA selection) should be added for Figures 2D and 2E, since the present analysis only describes the additive effect by FOA selection. While this comparison is informative to demonstrate the effect of the selection, it does not allow the comparison with WT cells (which I believe is the focus here).

While it would be interesting to make this comparison, we found it difficult to isolate and grow individual FOA resistant wild-type colonies in this experiment. We think that the

effect of selection is the key comparison here, as it indicates whether the enhanced *ura4+* silencing we observe correlates with increased H3K9me3 in the mutant strains and provides information about the silencing mechanism.

Finally, I also find that a table with all alleles used in this study would be helpful (point 1). The last point (#5) raised by this reviewer is interesting but I consider this as optional.

Addressed above
